# *trans*-Diaqua­bis­(dl-valinato-κ^2^ *N*,*O*)nickel(II)

**DOI:** 10.1107/S1600536811031072

**Published:** 2011-08-06

**Authors:** Amel Messai, Rim Benali-Cherif, Erwann Jeanneau, Nourredine Benali-Cherif

**Affiliations:** a Laboratoire des Structures, Propriétés et Interactions Interatomiques (LASPI^2^A), Centre Universitaire Abbes Laghrour–Khenchela, 40000 Khenchela, Algeria; b Université Claude Bernard Lyon 1, Laboratoire des Multimatériaux et Interfaces (UMR 5615), 69622 Villeurbanne Cedex, France

## Abstract

In the title complex, [Ni(C_5_H_9_NO_2_)_2_(H_2_O)_2_], the Ni^II^ atom, located on a centre of inversion, is *trans*-coordinated by two O atoms and two N atoms from d-bidentate valine and l-bidentate valine ligands and two water O atoms in an octa­hedral geometry. In the crystal, the discrete mononuclear units are linked into a three-dimensional network *via* O—H⋯O and N—H⋯O hydrogen bonds. C—H⋯O inter­actions are also observed.

## Related literature

For amino ­acids as ligands, see: Loo *et al.* (2005 ▶); Patrick *et al.* (2003 ▶). For valine, see: Ooiwa *et al.* (1995 ▶). For related complexes, see: Menabue *et al.* (1998 ▶)
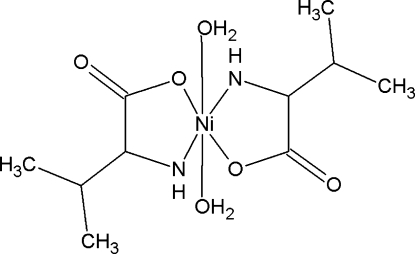


## Experimental

### 

#### Crystal data

[Ni(C_5_H_9_NO_2_)_2_(H_2_O)_2_]
*M* *_r_* = 325.01
Monoclinic, 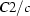

*a* = 24.8881 (2) Å
*b* = 5.8701 (3) Å
*c* = 10.0789 (2) Å
β = 90.442 (3)°
*V* = 1472.44 (8) Å^3^
*Z* = 4
Mo *K*α radiation
μ = 1.34 mm^−1^
*T* = 293 K
0.20 × 0.15 × 0.10 mm

#### Data collection

Nonius Mach3 KappaCCD diffractometer
2024 measured reflections
1960 independent reflections
1053 reflections with *I* > 2σ(*I*)
*R*_int_ = 0.023

#### Refinement

*R*[*F* ^2^ > 2σ(*F* ^2^)] = 0.053
*wR*(*F* ^2^) = 0.151
*S* = 1.03
1960 reflections
94 parameters
1 restraint
H-atom parameters not refined
Δρ_max_ = 0.48 e Å^−3^
Δρ_min_ = −1.26 e Å^−3^

### 

Data collection: *KappaCCD Server Software* (Nonius, 1998 ▶); cell refinement: *DENZO* and *SCALEPACK* (Otwinowski & Minor, 1997 ▶); data reduction: *DENZO* and *SCALEPACK*; program(s) used to solve structure: *SIR2004* (Burla *et al.*, 2005 ▶); program(s) used to refine structure: *SHELXL97* (Sheldrick, 2008 ▶); molecular graphics: *ORTEP-3* (Farrugia, 1997 ▶) and *PLATON* (Spek, 2009 ▶); software used to prepare material for publication: *WinGX* (Farrugia, 1999 ▶).

## Supplementary Material

Crystal structure: contains datablock(s) global, I. DOI: 10.1107/S1600536811031072/ds2128sup1.cif

Structure factors: contains datablock(s) I. DOI: 10.1107/S1600536811031072/ds2128Isup2.hkl

Additional supplementary materials:  crystallographic information; 3D view; checkCIF report

## Figures and Tables

**Table 1:** Hydrogen-bond geometry (Å, °)

*D*—H⋯*A*	*D*—H	H⋯*A*	*D*⋯*A*	*D*—H⋯*A*
N1—H1*N*⋯O2^i^	0.92	2.45 (3)	3.286 (5)	152
O1*W*—H1*W*⋯O1^ii^	0.95	1.74	2.684 (4)	172
O1*W*—H2*W*⋯O2^iii^	0.87	2.04	2.856 (5)	155
C5—H5*A*⋯O2^i^	0.96	2.53	3.483 (6)	170

Symmetry codes: (i) 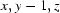; (ii) 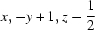; (iii) 
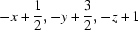
.

**Fig. 1.**
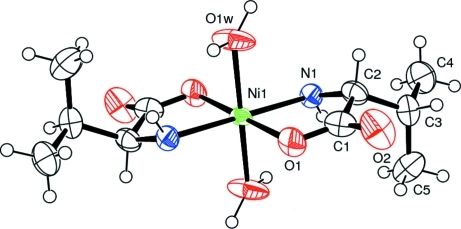
A view of the molecular structure of (I), showing the atom-numbering scheme and 30% displacement ellipsoids (arbitrary spheres for the H atoms)..

**Fig. 2.**
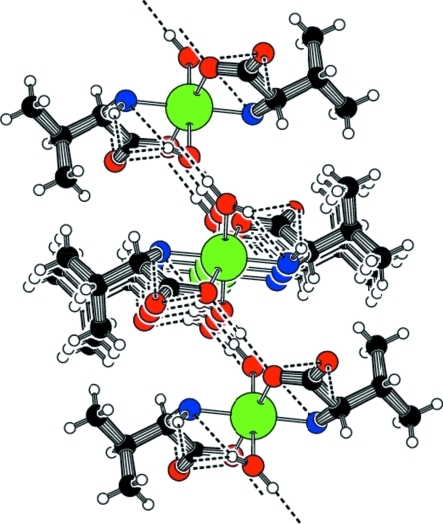
The packing for (I), viewed down [001], showing hydrogen bonds as dashed lines.

## supplementary crystallographic information

### Comment

Complexes of transition metals and amino acids have been extensively studied as models for the metal-binding sites in proteins. Amino acids are versatile ligands showing flexible coordination modes (Loo *et al.*, 2005) and they can coordinate to metal ions by their carboxylate and /or amino groups. Amino acid-metal complexes and their derivatives are of great importance because of their biochemical and pharmacological properties (Patrick *et al.*, 2003). Valine is an essential amino acid (Ooiwa *et al.*; 1995), and it can chelate to metal ions *via* its amino N atom and carboxylate O atom (Menabue *et al.*,1998). As a part of our studies on structural and properties of metal ion-amino acid complexes, we are reporting here the synthesis, crystal structure of a new Ni(DL-Val)_2_(H_2_O)_2_. The title compound is mononuclear, Ni(II) metal shows an octahedral geometry, it is in tans coordinated to D-bidentate valinate, L– bidentate valinate ions and two water molecules (Fig. 1). Each valinate ion chelates to the metal ion through its amino N atom and one of the carboxylate O atoms. The Ni—Oc (c = carboxylate, 2.019 (3) Å), Ni—Ow (w = water, 2.095 (3) Å) and Ni—N (2.087 (3) Å) bond distances agree well with published results for related complexes. The C—O bond of the noncoordinated carboxylate O atom [C1—O2 = 1.234 (5) Å] is only slightly shorter than the coordinated bond to the Ni ion [C1—O1 = 1.261 (6) Å], suggesting the involvement of atom O2 in hydrogen bonding, as described below. The crystal packing of (I) (Fig. 2) involves both N—H···O and O—H···O hydrogen bonds. The coordinated carboxylate O1 atom accepts an intermolecular hydrogen bond from the O1w water molecule. The non-coordinated atom O2 accepts hydrogen bonds from the O1w—H2w and N1—H1N1 groups of two different adjacent molecules. These interactions result in a two-dimensional network of hydrogen bonds.

### Experimental

To a hot solution (333 K) of guanidinoacetic acid (0.2342 g, 2 mmol) and DL-valine (0.2342 g, 2 mmol) in deionized water (100 ml) was slowly added a solution of nickel (II) nitrate (0.1827 g, 1 mmol) in deionized water (5 ml). The reaction mixture was stirred at 333 K for 8 h, cooled slowly to 277 K, and the pH adjusted to 6.0 with KOH (3 *M*). The white precipitate which formed was filtered off and the filtrate was stored in a covered vessel. Thin blue plate-like crystals began to be formed after the some weeks and were collected and washed with absolute ethanol and dried at 323 K.

### Refinement

The title compound crystallizes in the centrosymmetric space group C 2/c. All non-H atoms were refined with anisotropic atomic displacement parameters. H-atoms of water molecules and nitrohen were located in difference Fourier syntheses and not refined. Hydrogen atoms linked to carbon atoms were positioned geometrically and refined with a riding model, fixing the bond lengths at 0.98 and 0.96 A ° for CH and CH_3_ groups, respectively. The *U*_iso_(H) values were constrained to be 1.2Ueq (parent) or 1.5Ueq(methyl C).

### Crystal data

**Table:**

[Ni(C_5_H_9_NO_2_)_2_(H_2_O)_2_]	*F*(000) = 688
*M**_r_* = 325.01	*D*_x_ = 1.466 Mg m^−^^3^
Monoclinic, *C*2/*c*	Mo *K*α radiation, λ = 0.71073 Å
Hall symbol: -C 2yc	Cell parameters from 1960 reflections
*a* = 24.8881 (2) Å	θ = 3.3–29.1°
*b* = 5.8701 (3) Å	µ = 1.34 mm^−^^1^
*c* = 10.0789 (2) Å	*T* = 293 K
β = 90.442 (3)°	Placket, blue
*V* = 1472.44 (8) Å^3^	0.20 × 0.15 × 0.10 mm
*Z* = 4	

### Data collection

**Table:**

Nonius Mach3 KappaCCD diffractometer	1053 reflections with *I* > 2σ(*I*)
Radiation source: fine-focus sealed tube	*R*_int_ = 0.023
graphite	θ_max_ = 29.2°, θ_min_ = 3.3°
φ and ω scans	*h* = −31→16
2024 measured reflections	*k* = −4→7
1960 independent reflections	*l* = −13→10

### Refinement

**Table:**

Refinement on *F*^2^	Primary atom site location: structure-invariant direct methods
Least-squares matrix: full	Secondary atom site location: difference Fourier map
*R*[*F*^2^ > 2σ(*F*^2^)] = 0.053	Hydrogen site location: inferred from neighbouring sites
*wR*(*F*^2^) = 0.151	H-atom parameters not refined
*S* = 1.03	*w* = 1/[σ^2^(*F*_o_^2^) + (0.1031*P*)^2^] where *P* = (*F*_o_^2^ + 2*F*_c_^2^)/3
1960 reflections	(Δ/σ)_max_ < 0.001
94 parameters	Δρ_max_ = 0.48 e Å^−^^3^
1 restraint	Δρ_min_ = −1.26 e Å^−^^3^

### Special details

**Table:**

Geometry. All e.s.d.'s (except the e.s.d. in the dihedral angle between two l.s. planes) are estimated using the full covariance matrix. The cell e.s.d.'s are taken into account individually in the estimation of e.s.d.'s in distances, angles and torsion angles; correlations between e.s.d.'s in cell parameters are only used when they are defined by crystal symmetry. An approximate (isotropic) treatment of cell e.s.d.'s is used for estimating e.s.d.'s involving l.s. planes.
Refinement. Refinement of *F*^2^ against ALL reflections. The weighted *R*-factor *wR* and goodness of fit *S* are based on *F*^2^, conventional *R*-factors *R* are based on *F*, with *F* set to zero for negative *F*^2^. The threshold expression of *F*^2^ > σ(*F*^2^) is used only for calculating *R*-factors(gt) *etc*. and is not relevant to the choice of reflections for refinement. *R*-factors based on *F*^2^ are statistically about twice as large as those based on *F*, and *R*- factors based on ALL data will be even larger.

### Fractional atomic coordinates and isotropic or equivalent isotropic displacement parameters (Å^2^)

**Table:**

	*x*	*y*	*z*	*U*_iso_*/*U*_eq_	
Ni1	0.2500	0.2500	0.5000	0.0306 (3)	
O1	0.21580 (12)	0.5058 (5)	0.6033 (3)	0.0482 (7)	
O1W	0.26506 (15)	0.4700 (6)	0.3410 (3)	0.0706 (11)	
H1W	0.2466	0.4915	0.2588	0.106*	
H2W	0.2902	0.5473	0.3818	0.106*	
O2	0.14621 (18)	0.7348 (5)	0.6067 (4)	0.0693 (12)	
N1	0.17065 (13)	0.2015 (6)	0.4376 (3)	0.0363 (8)	
H1N	0.1516	0.0810	0.4722	0.044*	
C1	0.16898 (19)	0.5618 (7)	0.5671 (4)	0.0452 (10)	
C2	0.13961 (16)	0.4099 (7)	0.4637 (4)	0.0417 (9)	
H2	0.1387	0.4962	0.3805	0.050*	
C3	0.08064 (17)	0.3674 (8)	0.5024 (4)	0.0482 (10)	
H3	0.0632	0.5162	0.5115	0.058*	
C4	0.0511 (2)	0.2376 (9)	0.3929 (6)	0.0711 (17)	
H4A	0.0135	0.2290	0.4137	0.107*	
H4B	0.0656	0.0865	0.3861	0.107*	
H4C	0.0555	0.3157	0.3100	0.107*	
C5	0.0749 (2)	0.2437 (8)	0.6330 (5)	0.0662 (15)	
H5A	0.0899	0.0936	0.6255	0.099*	
H5B	0.0376	0.2325	0.6551	0.099*	
H5C	0.0936	0.3264	0.7013	0.099*	

### Atomic displacement parameters (Å^2^)

**Table:**

	*U*^11^	*U*^22^	*U*^33^	*U*^12^	*U*^13^	*U*^23^
Ni1	0.0303 (4)	0.0399 (4)	0.0215 (3)	−0.0096 (3)	−0.0074 (2)	0.0002 (3)
O1	0.0422 (16)	0.0578 (18)	0.0444 (15)	−0.0082 (14)	−0.0154 (13)	−0.0173 (14)
O1W	0.095 (3)	0.079 (2)	0.0377 (15)	−0.053 (2)	−0.0269 (16)	0.0222 (16)
O2	0.080 (3)	0.0384 (18)	0.089 (3)	0.0107 (16)	−0.022 (2)	−0.0196 (16)
N1	0.0307 (17)	0.0426 (19)	0.0356 (16)	−0.0079 (13)	−0.0048 (13)	−0.0087 (13)
C1	0.058 (3)	0.037 (2)	0.041 (2)	−0.010 (2)	−0.0073 (19)	−0.0010 (17)
C2	0.041 (2)	0.045 (2)	0.0385 (19)	−0.0071 (18)	−0.0112 (17)	0.0018 (17)
C3	0.037 (2)	0.045 (2)	0.063 (3)	0.0036 (18)	−0.004 (2)	−0.006 (2)
C4	0.044 (3)	0.099 (5)	0.070 (4)	−0.015 (3)	−0.018 (3)	−0.005 (3)
C5	0.066 (3)	0.074 (4)	0.058 (3)	−0.013 (2)	0.018 (3)	−0.013 (2)

### Geometric parameters (Å, °)

**Table:**

Ni1—O1	2.019 (3)	C1—C2	1.550 (5)
Ni1—O1^i^	2.019 (3)	C2—C3	1.542 (6)
Ni1—N1	2.087 (3)	C2—H2	0.9800
Ni1—N1^i^	2.087 (3)	C3—C5	1.511 (6)
Ni1—O1W^i^	2.095 (3)	C3—C4	1.525 (6)
Ni1—O1W	2.095 (3)	C3—H3	0.9800
O1—C1	1.262 (5)	C4—H4A	0.9600
O1W—H1W	0.9519	C4—H4B	0.9600
O1W—H2W	0.8736	C4—H4C	0.9600
O2—C1	1.231 (5)	C5—H5A	0.9600
N1—C2	1.472 (5)	C5—H5B	0.9600
N1—H1N	0.9209	C5—H5C	0.9600
			
O1—Ni1—O1^i^	180.00 (13)	N1—C2—C3	114.4 (3)
O1—Ni1—N1	81.68 (11)	N1—C2—C1	110.7 (3)
O1^i^—Ni1—N1	98.32 (11)	C3—C2—C1	111.6 (4)
O1—Ni1—N1^i^	98.32 (11)	N1—C2—H2	106.6
O1^i^—Ni1—N1^i^	81.68 (11)	C3—C2—H2	106.6
N1—Ni1—N1^i^	180.00 (6)	C1—C2—H2	106.6
O1—Ni1—O1W^i^	89.15 (15)	C5—C3—C4	110.0 (4)
O1^i^—Ni1—O1W^i^	90.85 (15)	C5—C3—C2	113.2 (4)
N1—Ni1—O1W^i^	88.38 (13)	C4—C3—C2	110.7 (4)
N1^i^—Ni1—O1W^i^	91.62 (13)	C5—C3—H3	107.6
O1—Ni1—O1W	90.85 (15)	C4—C3—H3	107.6
O1^i^—Ni1—O1W	89.15 (15)	C2—C3—H3	107.6
N1—Ni1—O1W	91.62 (13)	C3—C4—H4A	109.5
N1^i^—Ni1—O1W	88.38 (13)	C3—C4—H4B	109.5
O1W^i^—Ni1—O1W	180.0	H4A—C4—H4B	109.5
C1—O1—Ni1	115.9 (2)	C3—C4—H4C	109.5
Ni1—O1W—H1W	131.2	H4A—C4—H4C	109.5
Ni1—O1W—H2W	95.2	H4B—C4—H4C	109.5
H1W—O1W—H2W	132.9	C3—C5—H5A	109.5
C2—N1—Ni1	109.2 (2)	C3—C5—H5B	109.5
C2—N1—H1N	107.4	H5A—C5—H5B	109.5
Ni1—N1—H1N	118.6	C3—C5—H5C	109.5
O2—C1—O1	123.2 (4)	H5A—C5—H5C	109.5
O2—C1—C2	118.5 (4)	H5B—C5—H5C	109.5
O1—C1—C2	118.3 (4)		
			
O1^i^—Ni1—O1—C1	136 (100)	Ni1—O1—C1—C2	8.8 (5)
N1—Ni1—O1—C1	−15.6 (3)	Ni1—N1—C2—C3	−145.7 (3)
N1^i^—Ni1—O1—C1	164.4 (3)	Ni1—N1—C2—C1	−18.6 (4)
O1W^i^—Ni1—O1—C1	−104.1 (3)	O2—C1—C2—N1	−174.8 (4)
O1W—Ni1—O1—C1	75.9 (3)	O1—C1—C2—N1	7.4 (5)
O1—Ni1—N1—C2	18.4 (2)	O2—C1—C2—C3	−46.2 (5)
O1^i^—Ni1—N1—C2	−161.6 (2)	O1—C1—C2—C3	136.0 (4)
N1^i^—Ni1—N1—C2	−115 (70)	N1—C2—C3—C5	65.0 (5)
O1W^i^—Ni1—N1—C2	107.8 (3)	C1—C2—C3—C5	−61.6 (5)
O1W—Ni1—N1—C2	−72.2 (3)	N1—C2—C3—C4	−59.0 (5)
Ni1—O1—C1—O2	−168.9 (4)	C1—C2—C3—C4	174.4 (4)

Symmetry codes: (i) −*x*+1/2, −*y*+1/2, −*z*+1.

### Hydrogen-bond geometry (Å, °)

**Table:**

*D*—H···*A*	*D*—H	H···*A*	*D*···*A*	*D*—H···*A*
N1—H1N···O2^ii^	0.92	2.45 (3)	3.286 (5)	152
O1W—H1W···O1^iii^	0.95	1.74	2.684 (4)	172
O1W—H2W···O2^iv^	0.87	2.04	2.856 (5)	155
C5—H5A···O2^ii^	0.96	2.53	3.483 (6)	170

Symmetry codes: (ii) *x*, *y*−1, *z*; (iii) *x*, −*y*+1, *z*−1/2; (iv) −*x*+1/2, −*y*+3/2, −*z*+1.
